# Alginate Microcapsules as Nutrient Suppliers: An *In Vitro* Study

**DOI:** 10.22074/cellj.2018.4508

**Published:** 2018-01-01

**Authors:** Ahad Khoshzaban, Peyman Keyhanvar, Elham Delrish, Farhood Najafi, Saeed Heidari Keshel, Ikuya Watanabe, Alireza Valanezhad, Tahereh Jafarzadeh Kashi

**Affiliations:** 1 Iranian Tissue Bank and Research Center, Imam Khomeini Medical Complex Hospital, Tehran University of Medical Sciences, Tehran, Iran; 2 Stem Cell Preparation Unit, Farabi Eye Hospital, Tehran University of Medical Sciences, Tehran, Iran; 3 Department of Dental Biomaterials, School of Dentistry, Tehran University of Medical Sciences, Tehran, Iran; 4 Department of Resin and Additives, Institute for Color Science and Technology, Tehran, Iran; 5 Department of Bio-Engineering, Nagasaki University, Nagasaki, Japan

**Keywords:** Alginate, Cell Culture, Cell Viability, Growth Media, Microcapsule

## Abstract

**Objective:**

Alginate, known as a group of anionic polysaccharides extracted from seaweeds, has attracted the attention of researchers because of its biocompatibility and degradability properties. Alginate has shown beneficial effects on wound healing as it has similar function as extracellular matrix. Alginate microcapsules (AM) that are used in tissue engineering as well as Dulbecco’s modified Eagle’s medium (DMEM) contain nutrients required for cell viability. The purpose of this research was introducing AM in medium and nutrient reagent cells and making a comparison with control group cells that have been normally cultured in long term.

**Materials and Methods:**

In this experimental study, AM were shaped in distilled water, it was dropped at 5 mL/hours through a flat 25G5/8 sterile needle into a crosslinking bath containing 0.1 M calcium chloride to produce calcium alginate microspheres. Then, the size of microcapsules (300-350 µm) were confirmed by Scanning Electron Microscopy (SEM) images after the filtration for selection of the best size. Next, DMEM was injected into AM. Afterward, adipose- derived mesenchymal stem cells (ADSCs) and Ringer’s serum were added. Then, MTT and DAPI assays were used for cell viability and nucleus staining, respectively. Also, morphology of microcapsules was determined under invert microscopy.

**Results:**

Evaluation of the cells performed for spatial media/microcapsules at the volume of 40 µl, showed ADSCs after 1-day cell culture. Also, MTT assay results showed a significant difference in the viability of sustained-release media injected to microcapsules (P<0.05). DAPI staining revealed living cells on the microcapsules after 1 to 7-day cell culture.

**Conclusion:**

According to the results, AM had a positive effect on cell viability in scaffolds and tissue engineering and provide nutrients needed in cell therapy.

## Introduction

Alginate is a popular group of anionic polysaccharides extracted from seaweeds (1, 2) that could be produced by some brown algae and certain bacteria species such as *Azotobacter vinelandii* and *Pseudomonas aeruginosa* (2). Also, alginate has different applications and could be found in nature in the forms heteropolysaccharide hydrogel of ß-D-mannuronate and a-L-guluronate, physically cross-linked with divalent ions such as calcium to form an anionic hydrogel (1, 3, 4). The simple, mild aqueous-based gel formation of sodium alginate in the presence of divalent cations is suitable for encapsulation of various drugs with different properties (3). 

The scaffold usage is justified as it provides a suitable headstock for cell growth, proliferation and differentiation (2). *In vitro* and *in vivo* studies revealed that no scaffold can guarantee long-term viability of cells. Because of biocompatible, non-toxic and non-immunogenic properties of alginate, it is used as a common scaffold which functions both as a cytokines carrier and scaffold (4). In addition, it can be used in capsule form as a carrier of the stem cells (3). One of the applications of this material is protection of stem cells from immune responses (4). Therefore, the nutrient release and cytokines properties of alginate can be influenced its clinical application particularly in bone tissue engineering and vascularization (2, 3). 

Alginate microcapsules (AM) can be saturated with different solutions (3) and they may have direct effects on cells. It is reported that AM helps the smart differentiation of stem cells and induced pluripotent stem (IPS) cells. It is reported that alginate can be used for stem cells encapsulation (5). 

The microcapsules have disadvantages such as being easily ruptured as they possess low micromechanical properties. Up to the present, several studies have been done on application of different materials or changing the synthesis protocols to improve the micromechanical properties of AM (4). For instance, CaCl_2_-anionic hydrogel-chitosan, G (a-L-guluronic acid) and M (ß-D-mannuronic acid) were used to enhance the micromechanical properties of AM (1, 3). 

The molecular composition of alginates depends on the organism and isolated tissue by which the alginate has been produced (3). Alginates prepared from the stipes of old *L. Hyperborea* kelp contain the highest content of *a-Lguluronic acid* residues while alginate from *Ascophyllum nodosumand Lonicera japonica*, has lower amounts of a-L-guluronic acid (6). It is revealed that alginate is not subjected to a regular repeatability according to Bernoullian statistics (i.e. the units of alginate are found in a separate chain and it has monomers along) (3). In cell therapy, the optimum time within which cells should be viable is at least 72 hours and alginate is expected to have the capacity of maintaining cell viability for this period (7). In most studies, AM is used for stem cell maintenance and differentiation (5-7). Also, alginate hydrogel accelerates wound healing process and prevents infection (8).

According to AM medical activity or alginate hydrogel, the supply of nutrient solution has been used in cell culture (8). Nutrients (cell culture media) have been frequently used in cell culture but clinical investigations indicated that cells injection might not work in the absence of nutrients (7). The main objective of the present study is application of microcapsules as suppliers of nutrients for growth and proliferation of cells in comparison with conventional culture method. 

## Materials and Methods

### Alginate microcapsules synthesis 

**Fig.1:**
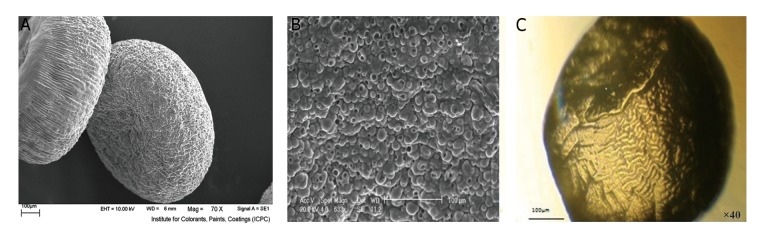
Alginate microcapsule (AM) scanning electronic and microscopic views. A. Two AM in scanning electron microscopy (SEM) view (×70), B. AM view in SEM view (20 KV), and C. One microcapsule under invert microscope (×40).

In this experimental study, sodium alginate, potassium and calcium chlorides were procured from Sigma Aldrich, UK. The chemicals were used without further purification. The particle size and surface morphology of the microcapsules were examined by Scanning Electron Microscopy (SEM) (Fig .1A, B). 

### Fabrication of microspheres

The AM were typically synthesized as previously described (6). A total of 3.1 g of alginate was added to 100 mL of distilled water and the resultant mixture was stirred until the alginate was completely dissolved. Afterward, the alginate solution was filtered and passed through a syringe pump to form droplets. Then, it was dropped at 5 mL/hour through a flat 25G5/8 sterile needle into a crosslinking bath containing 0.1 M calcium chloride to produce calcium alginate microspheres (6-8). To reduce droplets size, airflow of 12.5 L/minute through 3 mm tube was used over the needle. The alginate droplets were transformed to alginate beads by gelling in a 100 mM CaCl_2_ solution (containing 2 mM KCl) for at least 10 minutes. Subsequently, the beads were washed three times in Ca^2+^-, Mg^2+^-free solution (GibcoBRL) (pH=7.0) containing 150 mM of NaCl (Merelbeke, Belgium). The capsules were suspended for another 3 minutes in a 0.3% solution of alginate. As a result, the capsules had a diameter of 300 µm (Fig .1A).

### Particle size test

For selection of the best size of microcapsules, diffraction particle size analyzer (0.03-1000 µm) SALD2201 laser (Shimadzu, Japan) was used. According to ISO 13320 (9) and USP (10), this method is suitable for micron-sized polymer capsules such as alginate. The obtained graph showed size variation and filtration which was used to select the best size.

### Nutrient load in the microcapsules

**Fig.2:**
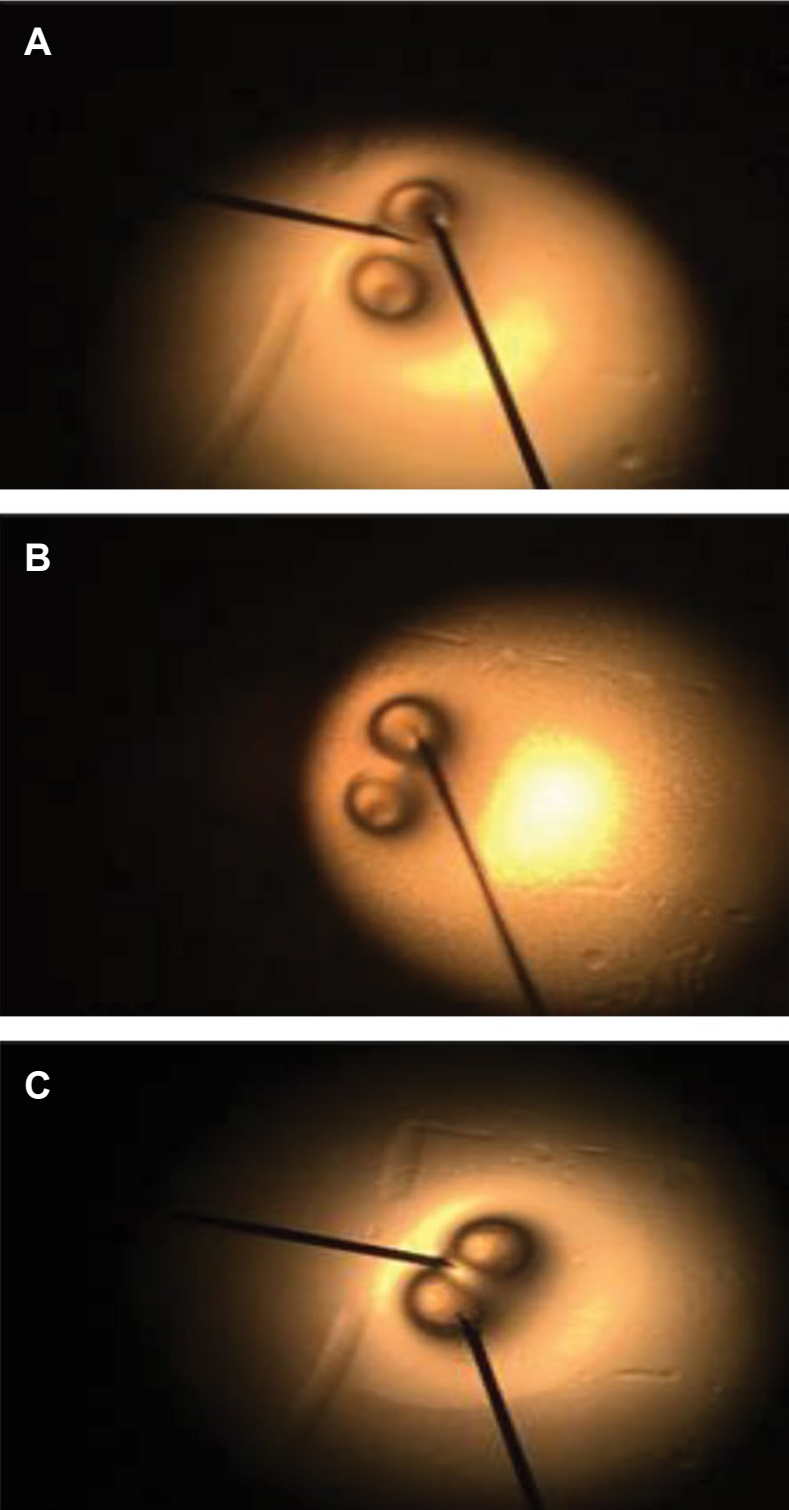
Injection media into microcapsules. A. Penetration of needle (32 G) in alginate microcapsule (AM), B. Filling procedure, and C. AM filled with nutrient (DMEM).

After synthesis of microcapsules, they were transferred and pictures were taken under inverted florescent microscope (CTI, Spain) (Fig .1C). Then, 10 µL of the nutrient media containing DMEM low glucose (Gibco, USA) and 10% fetal bovine serum (FBS, Gibco, USA) was loaded by super fine needles (Aesculap, Germany) (Fig .2).

### Releasing test

Micro-capsules were weighed before and after injection of cell culture media (on day 1, 2 and 10). Microcapsules’ weight was equivalent to the weight of the empty microcapsules on day 10.

### Adipose-derived mesenchymal stem cell isolation, identification and culture 

Adipose-derived mesenchymal stem cells (ADSCs) were isolated by enzymatic digestion according to Park AM protocol. Briefly, lipoaspirate tissues (25 ml) were taken from two volunteers and washed with PBS in a 50ml Falcon tube. The tissues were then digested with an equal volume of 0.2% collagenase type IV at 37°C for 15 minutes, and the stromal vascular fraction cells were isolated by centrifugation at 300 g at room temperature. Viable cells (1×10^6^) were cultured in 75-cm^2^ flasks in 10% FBS-supplemented medium (DMEM/F 12). After 2 days, the unattached cells were discarded by replacing the medium with fresh medium. The medium was subsequently changed twice a week. At 80 to 90% confluence, both types of cells were harvested with trypsin- ethylene diamine tetra acetic acid (EDTA, Gibco, UK) and subsequently replated at 2000 cells/cm^2^. For analysis of surface markers expression, at passage 3, AMSCs were washed three times with phosphate-buffered saline (PBS), then incubated with a blocking solution of 3% serum in PBS for 30 minutes. After centrifugation, 5×10^5^ cells were suspended in blocking solution, then incubated with antibodies against human CD31, CD166, CD90, CD44, CD73, CD34 and CD 45 (Abcam, UK). After incubation for 30 minutes, the cells were washed with PBS, then analysis was made by using FACSCalibur flow cytometer (Becton Dickinson, San Jose, CA, USA) according to the standard procedures (Figes.3, 4). 

**Fig.3:**
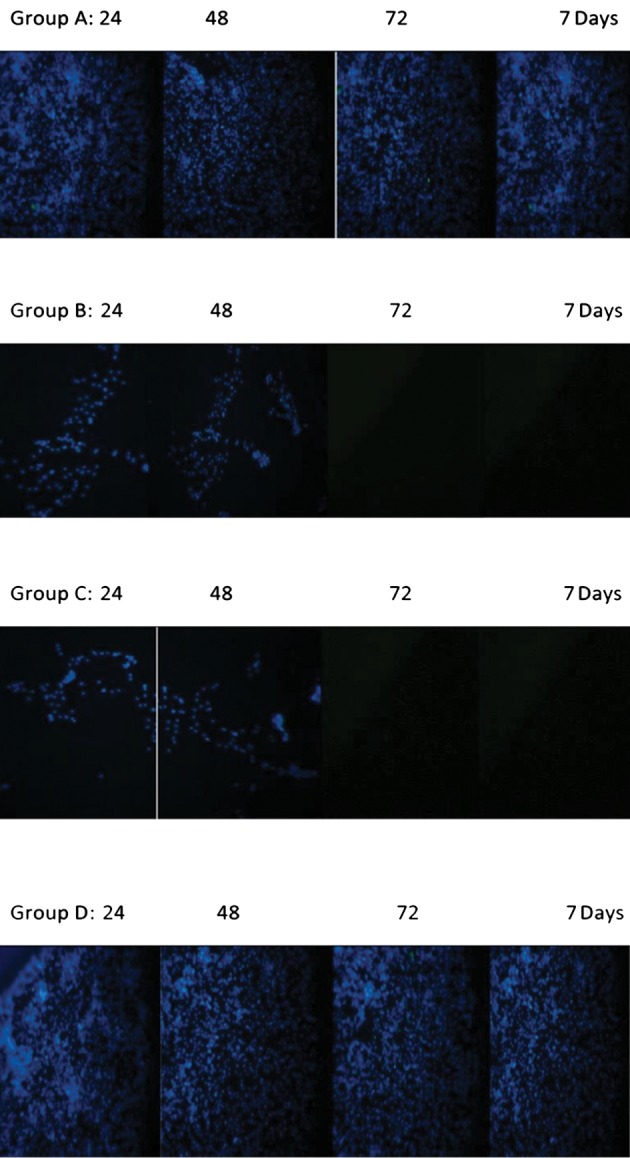
DAPI staining cells at different time points in 4 groups. In groups B and C dark fields were observed after 72 hours and 7 days (CTI Microscope, Spain, ×20).

**Fig.4:**
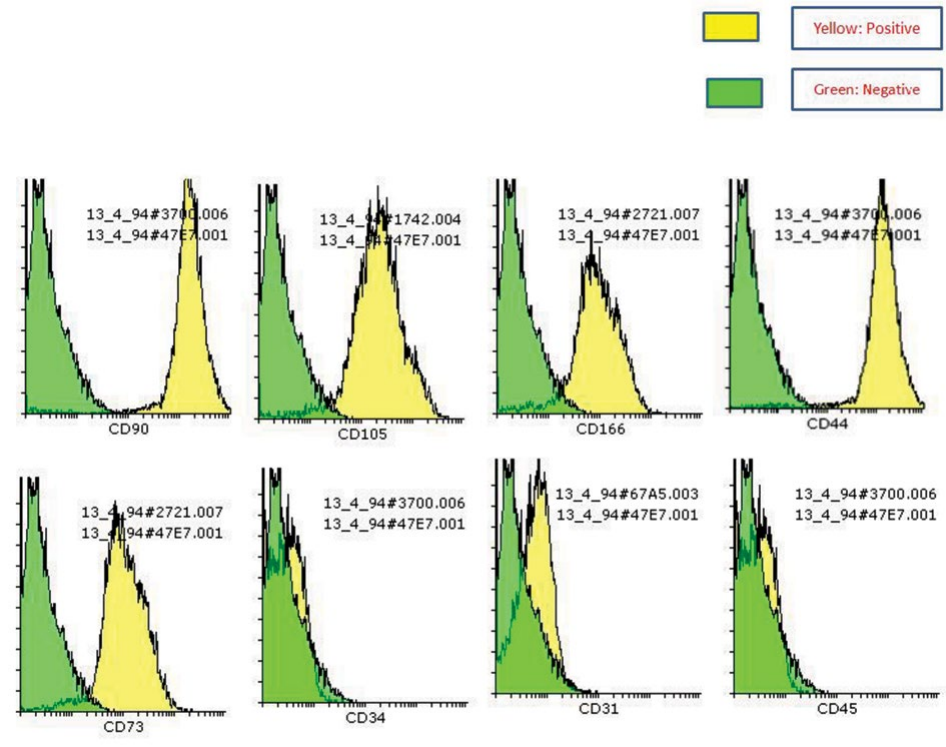
Adipose-derived mesenchymal stem cells flow cytometry.

### Viability cells adhering by microcapsules and nutrient

After loading the microcapsules, the experimental cells (Human Adipose-Derived Mesenchymal Stem Cells, AMS) were used in Stem Cell Preparation Unit Lab, Farabi Eye Hospital, Tehran, Iran. All information concerning this cell line was recorded in Farabi Eye Hospital.

### Experimental and control groups

The cells were cultured 96-well plates (Falcon, USA), at the volume of 40 µl. Four experimental groups were used to determine the role of AM in cell contact and viability in cell therapy as compared to nutrient media. Group A (positive control): AMS cells treated with standard nutrient media 40 µl+20 µl [DMEM low glucose and 10% FBS Gibco, USA]. Group B (experimental control): AMS cells (40 µl) with Ringer’s serum (20 µl, Razi Co, Iran). Group C: AMS cells (40 µl) with empty AM+Ringer’s serum. Group D: AMS cells (40 µl) with AM+standard nutrient media+Ringer’s serum (20 µl).

### MTT analysis

Viability of AMSCs grown on AM was assessed by using 3,4,5 dimethylyhiazol-2yl-2-5 diphenyl tetrazolium bromide (MTT) assay according to the protocol of MTT. Cells that were grown in normal media without AM served as a control for the interpretation of data. Then, 4 wells were selected from 96 wells for each group. Totally from 4 groups, 16 wells were filled. For each group 20 µl of MTT solution was added, then the plates were transferred to a CO_2_ incubator (Sanyo, Japan). After 4 hours, the liquid was removed and 100 µl dimethyl sulfoxide (DMSO) was added. Finally, each of 96-well was transferred to the ELISA reader (Bio-Rad, USA). Then, the plates were read at 570 nm and the reference optical density (OD) was 600 nm. For each plate, MTT assay was done at 24, 48, and 72 hours and on day 7.

### DAPI staining

The DAPI assay was done based on the following steps. 4’, 6’ diamino-2-phenylindole 2HCl was used for specific staining at pH=7. In order to stain, each well was fixed using 60 µL paraformaldehyde (PFA) 4% for 8 minutes. The PFA should be dumped onto paper towels and wrapped in aluminum foils before disposal. Then, cells were washed with 60 µL/well of 1X PBS, for 3 times each time for 5 minutes. Cells were mobilized using 60 µL/well of 0.1% Triton-X-100 for 10 minutes and this process was repeated for 3 times. Cells were stained with 50 µL/well of DAPI (1:2000 dilution, in 1X TBST) for 5 minutes and 50 µL/well of 1X PBS was added to keep the cells hydrated while imaging on the Image Express Micro (Fig .3).

### Statistical analysis

The results are presented as mean ± SD. The statistical differences were analyzed by one -way ANOVA followed by Dunnett’s tests. A P<0.05 was considered to be significant. All assays were performed in quadruplicate.

## Results

### MTT analysis

**Fig.5:**
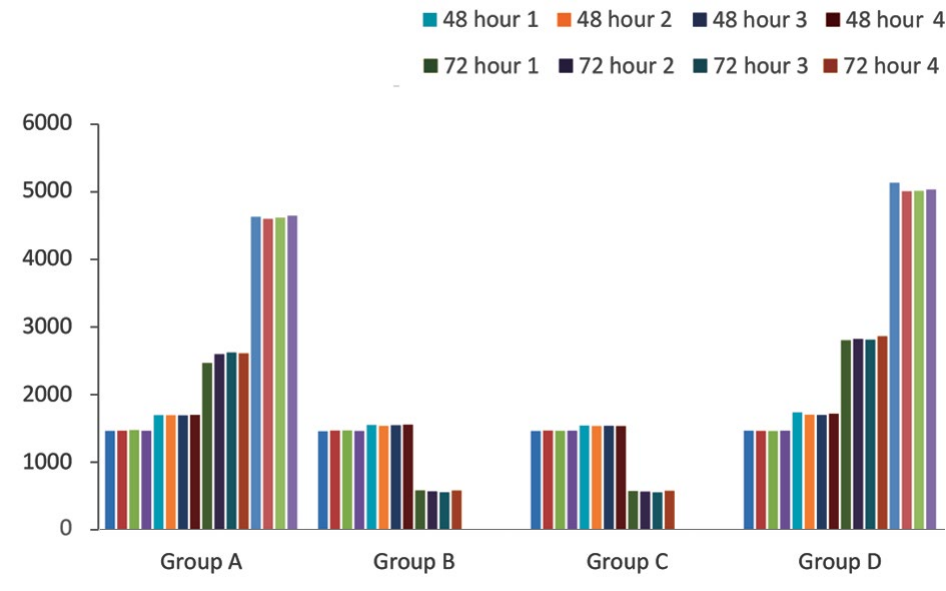
MTT Assay results for each group (4 wells per group). Group B and C had no viable cells after 7 days.

Group A had a cell viability of 95% ± 3 at 24, 48, and 72 hours and on day 7. In groups B and C, after 24 hours, the number of viable cells reduced; therefor, after day 7, particularly viable cells were not seen (viable cells or carcass were 0% ± 3). Group D showed the same results as group A. Accordingly, there were no significant differences between groups A and D (P>0.05). A significant difference was observed in groups B and C compared to group D (P<0.05, Fig .5).

### Particle size analysis

**Fig.6:**
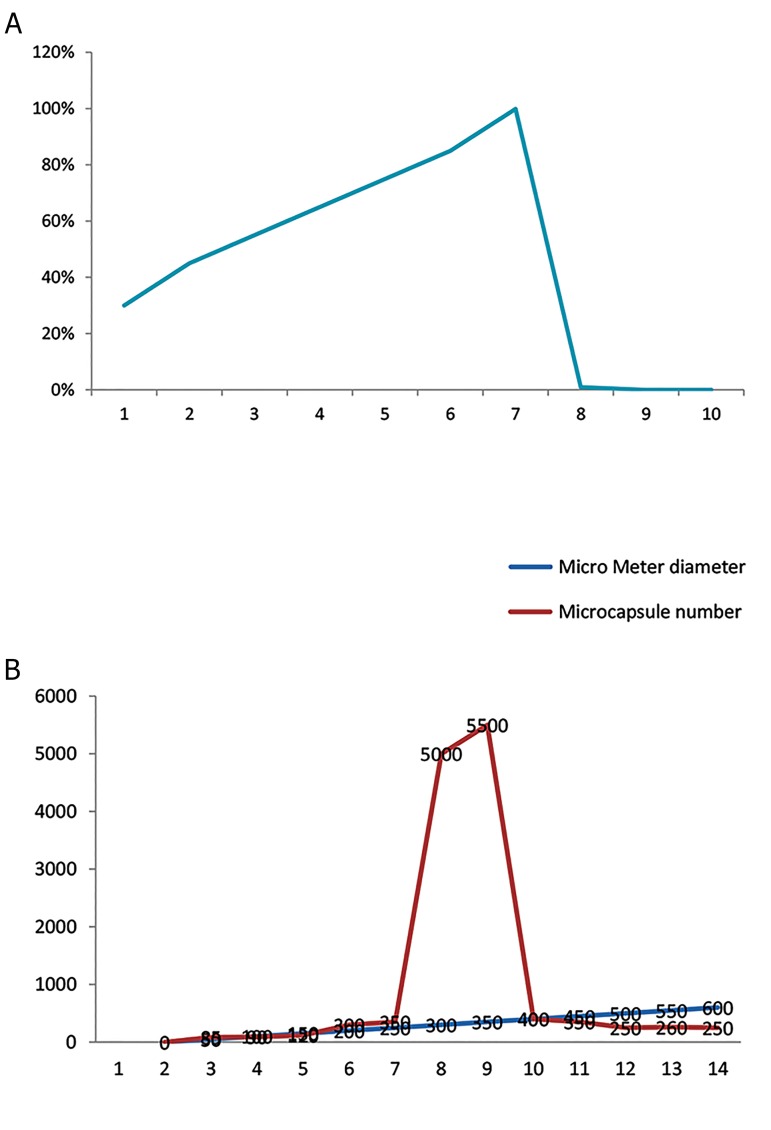
Diagrams of particle size and releasing time of microcapsules. A. Microcapsules particle size and B. Linear diagram of alginate microcapsules releasing time.

Microcapsules within the size range of 300-350 µm were selected and used as the maximum size of microcapsules (Fig .6A).

### DAPI staining

According to our data, the cells in groups A and D were stained the same at 24, 48, 72 hours and on day 7 with high viability. Accordingly, groups B and C did not differ significantly and showed less viability than that of groups A and D particularly after 24 hours. However, considerable differences were detected at 72 hours and on day 7 day (Fig .4).

### Releasing test

The reliable time was in day 7, on which the media was totally released (Fig .6B).

## Discussion

In several studies, AM have been used as a supplier of nutrients for human fatty-derived mesenchymal stem cells. Alginates are polyanionic copolymers which have ionic interactions between the guluronic acid groups (6-9). The standard and effective substrate used for cell growth in cell culture is DMEM which is widely used for the growth of different mammalian cells (7). On the basis of different reports, many cells such as primary fibroblasts, neurons, glial cells, HUVECs and smooth muscle cells, as well as cell lines like HeLa, 293, and Cos-7 were effectively cultured in DMEM (8). 

DMEM was originally formulated with low glucose (1 g/L) and sodium pyruvate, but it is often used with higher glucose levels, with or without sodium pyruvate for cell differentiation. Gibco DMEM with GlutaMAX™ supplement minimizes toxic ammonia buildup and improves cell viability and growth (10-12). Sodium alginate is biologically safe and widely used in drug delivery systems (11). It is used to encapsulate various drugs in alginate beads (13, 14) and to belay matrix beads (15, 16). Controlling the release of dosage maintains a consistent therapeutic level of drug and minimizes the adverse effects. This is suitable for drug therapy as it can prolong the release of the drug over an extended period, reduce the frequency of administration, and increase patient compliance (17, 18).

Moshaverinia et al. (19) used the injectable oxidized alginate micro beads which encapsulated periodontal ligament (PDL) and gingival mesenchymal stem cells. As observed in the current study, AM were filled with DMEM using very fine needles. Then, they were added to mesenchymal stem cells. The null hypothesis was that MA might release nutritional media into cell environment and maintain the viability of stem cells for a short or long period. In the present research, MTT assay and DAPI staining were used to monitor adipose-derived mesenchymal stem cells (ADSCs) cell culturing. According to our data, similar results were detected in groups A and D. However, cells in groups B and C were visible after 24, 48, and 72 hours and after 7 days. The findings of the current study can be used as the basic information which can open a new window for alginate usage. 

There is an increasing interest in microcapsules due to their wide applications in biomedicine (20), bioreactors, therapeutics, drug delivery system and tissue engineering (21). Among several materials available for production of microcapsules, the most commonly used microsphere is alginate (19, 22). Researchers have focused on stimuli- responsive polymeric-hydrogels using alginate due to its potential applications in drug delivery systems and tissue engineering (23-27). Lindenhayn et al. (28) used AM for cartilage tissue engineering and it was detected that AM protect stem cells. This effect is mediated by double-membrane microcapsules with a multi-enzyme system through self-assembly and bio-mineralized properties (26-29). 

In the present study, AM (beads) were prepared as a reservoir containing nutrient media (DMEM) which was injected to AM. This method is noble and may be applicable in *in vitro* and *in vivo* studies or in clinical trials of cell therapy. The MTT and DAPI results showed that AM containing DMEM could support the cells for 7 days. The obtained results can be used as basic information and future studies should be conducted to find direct and cellular mechanism(s) underlying the protective properties of AM on stem cells. Perhaps this technique can improve cell culture and cell therapy to be ultimately used in animal studies or clinical trials. 

## Conclusion

This achievement may open new horizons and create new approach for cell viability maintained on new condition of cell/stem cell culturing media (AM/ media) with sustain release and good biocompatibility manner without refeeding consecutive. This may lead to protraction of cell viability on scaffold either in animals or humans body. The fruition of this survey can play vital role in the application of AM in tissue engineering and acts as a supplementary factor for nutrition 72 hours after implantation of scaffolds with cells in body. It can also be used as nutrient reservoir or nutrient pump or the carrier for cell differentiation cytokine in modern applied techniques. Briefly, nutrition media release of AM showed effective results. This study can create a new approach on the application of the AM in tissue engineering and cell transporting in animal or clinical trials.
